# Complete Genome Sequence of *Flavobacterium* *psychrophilum* Strain OSU THCO2-90, Used for Functional Genetic Analysis

**DOI:** 10.1128/genomeA.01665-16

**Published:** 2017-02-23

**Authors:** Tatiana Rochat, Paul Barbier, Pierre Nicolas, Valentin Loux, David Pérez-Pascual, José A. Guijarro, Jean-François Bernardet, Eric Duchaud

**Affiliations:** a VIM, INRA, Université Paris-Saclay, Jouy-en-Josas, France; b MaIage, INRA, Université Paris-Saclay, Jouy-en-Josas, France; c Área de Microbiología, Departamento de Biología Funcional, Facultad de Medicina, Instituto de Biotecnología de Asturias, Universidad de Oviedo, Oviedo, Spain

## Abstract

We report here the complete annotated genome sequence of *Flavobacterium psychrophilum* OSU THCO2-90, isolated from Coho salmon (*Oncorhynchus kisutch*) in Oregon. The genome consists of a circular chromosome with 2,343 predicted open reading frames. This strain has proved to be a valuable tool for functional genomics.

## GENOME ANNOUNCEMENT

*Flavobacterium psychrophilum* is a member of the family *Flavobacteriaceae*, phylum *Bacteroidetes*. This bacterium, initially reported from North America, is now documented worldwide (1–5) and is currently one of the most devastating bacterial pathogens of farmed salmonids reared in freshwater (6). The two main clinical forms are rainbow trout fry syndrome and bacterial coldwater disease (7). As a complement to the *F. psychrophilum* genomes already available (8–10), we report here the complete genome sequence of strain OSU THCO2-90 (11). This strain was isolated by R. A. Holt (Oregon State University) from the kidney of a Coho salmon (*Oncorhynchus kisutch*) in Oregon in 1990. Its sequence type is ST9 (3), and it is moderately virulent in a rainbow trout experimental infection model using injection (12) or bath challenge (unpublished). Importantly, it is so far the only *F. psychrophilum* strain that can be successfully genetically manipulated (13, 14). Indeed, this strain has proved to be a valuable tool for functional genomics and has already contributed to the characterization of several genes involved in pathogenicity (12, 15–17).

Sequencing used a combination of Sanger (ABI3730, Applied Biosystems), 454 (GS-FLX, Roche), and Solexa (GAIIx) sequencing with 2.5-fold, 17-fold, and 75-fold coverage, respectively. The 454 reads were assembled into 186 contigs (>500 bp) and 30 scaffolds using Newbler (Roche). These contigs and the Sanger reads were assembled using Phrap (18). Gaps were closed using primer walking on pCNS clones (10-kb fragments on average) used for Sanger sequencing or by PCR sequencing; Solexa reads were used to correct residual sequencing errors. The genome was closed to a single chromosome and the assembly was validated by optical mapping using *Nco*I (19). Genome annotation was performed using the AGMIAL annotation platform and then manually validated and enriched (20).

The genome of *F. psychrophilum* OSU THCO2-90 consists of a circular chromosome of 2,783,852 bp with an overall G+C content of 32.61%. The genome is predicted to encode 2,343 protein-coding genes, 49 tRNA genes, and six rRNA operons.

*F. psychrophilum* OSU THCO2-90 belongs to the same clonal complex as the type strain ATCC 49418, which was also isolated from Coho salmon but differs from isolates retrieved from rainbow trout, thus demonstrating host specificity (3). Strains OSU THCO2-90 and ATCC 49418^T^ share 2,259 protein-coding genes (≥80% protein identity; 80% protein overlap). The most striking differences between these two strains are (i) a bona fide CRISPR locus encompassing 42 direct repeats in strain OSU THCO2-90, whereas strain ATCC 49418^T^ contains only a remnant CRISPR system; (ii) a genomic island in strain OSU THCO2-90 (genomic coordinates: 1,323,198 to 1,398,091); and (iii) the absence of a 2.7-kb plasmid in strain OSU THCO2-90 (19), which probably makes it more amenable to genetic manipulations (13).

The availability of this complete genome may help in understanding host specificity and genome evolution and will facilitate functional genomics studies. Using Tn*4351*-mediated random mutagenesis, we already obtained about 1,000 mutants whose precise sites of transposon integration into the chromosome can easily be determined by inverse PCR (15).

### Accession number(s).

This genome has been deposited in ENA under accession number LT670843.
